# Evaluation of Nutritional Status During Induction Chemotherapy in Patients With Acute Leukemia

**DOI:** 10.7759/cureus.83547

**Published:** 2025-05-05

**Authors:** Steve Thomas, Manoranjan Mahapatra, Tulika Seth, Jyothsna Viveka

**Affiliations:** 1 Hematology, Sri Ramachandra Institute of Higher Education and Research, Chennai, IND; 2 Hematology, All India Institute of Medical Sciences, New Delhi, IND; 3 Endocrinology and Diabetes, All India Institute of Medical Sciences, New Delhi, IND

**Keywords:** acute leukemia, induction chemotherapy, malnutrition, poor prognosis, undernutrition

## Abstract

Background: Malnutrition is often prevalent in most of the children living in resource-limited countries. This study aimed to evaluate the occurrence of malnutrition and micronutrient deficiency in patients with acute leukemia undergoing induction chemotherapy and to report the various outcomes regarding complications and remission rates.

Materials and methods: This prospective observational study was conducted at the hematology wards of All India Institute of Medical Sciences (AIIMS), New Delhi from February 2018 to August 2019. The study involved children and adolescents aged two to 20 years, of any gender, diagnosed with acute leukemia, and undergoing induction chemotherapy. Baseline characteristics were recorded along with nutritional parameters like micronutrients at admission, two weeks, and four weeks (at the end of induction).

Results: A total of 64 patients were enrolled, and the majority of them were males (67.18%). Based on body mass index (BMI), patients were categorized into underweight (45.31%), healthy weight (51.56%), and overweight (3.13%) groups. B-cell acute lymphoblastic leukemia (B-ALL) was the most common diagnosis. There was a noticeable trend of worsening undernutrition in both underweight and healthy weight groups. The mean BMI decreased significantly from 16.71 kg/m^2^ at admission to 16.05 kg/m^2 ^and 16 kg/m^2 ^at weeks two and four, respectively (P=0.0001). Serum albumin levels at admission and after two weeks showed a strong correlation with mortality (P<0.001) and a poor prognosis (P<0.001). Serum iron levels dropped significantly from 165.5 ng/mL (on admission) to 126 ng/mL at two weeks (P=0.001), and total iron binding capacity (TIBC) values also decreased from 231.5 mcg/dL to 195.5 mcg/dL at two weeks (P=0.04). Additionally, the underweight category experienced higher morbidity compared to the healthy weight group (65.52% vs. 39.39%; P=0.043).

Conclusion: The study highlights the critical importance of assessing and addressing the nutritional status of acute leukemia patients upon admission, as undernourished individuals exhibited higher morbidity and required more chemotherapy dose adjustments.

## Introduction

Acute lymphoblastic leukemia (ALL) stands as the most prevalent form of cancer in children, comprising around 25% of all cases of childhood malignancy [1]. Lower survival and lower cure rates of childhood and adolescent acute leukemia in developing countries compared to the developed ones is a well-known fact [2]. According to data from the population-based cancer registries in Bengaluru and Chennai, the recovery rate for leukemia is significantly lower in India. This could be attributed to a variety of factors such as restricted access to proper treatment, limited resources, delayed diagnosis, infections, underlying malnutrition, poor compliance to therapy, and lack of adequate supportive care [3]. Mortality in the early stages of treatment poses a significant challenge and stands as a primary barrier to enhancing survival rates among children and adolescents with acute leukemia in developing nations. The majority of these fatalities can be attributed to infections and a lack of tolerance for the treatment regimen. Additionally, there is a higher proportion of patients classified as high risk with advanced disease, in contrast to those classified as low risk.

Undernutrition refers to a state characterized by inadequate nutritional status, arising from an imbalance between the energy and nutrients supplied and the body's requirements [4]. The occurrence of undernutrition among children and adolescents receiving cancer treatment has been reported to range widely from 0% to 70% [5]. The nutritional status can significantly impact various clinical outcomes, including overall survival and event-free survival, treatment tolerance, susceptibility to infections, and overall quality of life [6]. An advanced stage of cancer results in a modified nutritional condition characterized by a deficiency in both protein and micronutrients. The importance of nutrition in children and adolescents with cancer is still an underestimated topic within hematology. It is recognized that a diminished nutritional status may contribute to poor immune function, disrupt drug metabolism, lead to drug toxicities, and result in adverse clinical outcomes [7,8]. Numerous studies conducted in developed nations have examined how malnutrition affects the outlook and survival of children with acute leukemia, with a prevailing belief that malnutrition plays a significant role in patient survival. However, it is crucial to ascertain whether a similar correlation exists in developing countries, where malnutrition is widespread, and there is a higher rate of mortality during the initial phase of treatment [9,10]. The reduced survival rate of childhood ALL in developing countries has been linked to undernutrition, an increased risk of infections, insufficient access to proper supportive care, and inadequate adherence to therapy [2]. There was a noteworthy decrease in folate levels observed in consecutive assays during chemotherapy [11]. Children with folate deficiency faced an elevated risk of experiencing delayed marrow recovery and lower blood counts; additionally, hypoalbuminemia, vitamin B12 deficiency, and folate deficiency were all linked to an increased likelihood of toxic deaths occurring during the induction phase [12]. The proportion of undernourished children with ALL varies from nearly 10% in the developed nations [2] to more than 60% in the developing countries [13]. Low folate and B12 levels are common in Indian children [14,15], and lower levels in children with ALL have been earlier reported [16].

To address this issue, the present study aimed to evaluate the occurrence of malnutrition and micronutrient deficiency in patients with acute leukemia who are admitted for induction chemotherapy and to report the various outcomes in regard to complications and remission rates.

## Materials and methods

Study design

This prospective, observational study was conducted at hematology wards in All India Institute of Medical Sciences (AIIMS) New Delhi over a period of 18 months between February 2018 and August 2019. The study was approved by the institutional ethics committee and was performed in accordance with the Declaration of Helsinki and the International Conference on Harmonization guidelines. Written informed consent was obtained from all the participants prior to enrollment in this study.

Study participants

The study included children and adolescents (aged two to 20 years) of either sex with a diagnosis of acute leukemia (ALL/acute myelogenous leukemia (AML)) or undergoing induction chemotherapy in hematology wards. Those who have already received partial treatment from elsewhere and have relapsed disease were excluded from the study.

Data collection

Baseline characteristics such as height, weight, body mass index (BMI), and percentiles were recorded along with nutritional parameters like micronutrients(Ca, P, Mg, Vitamin D, Vitamin B12, folate, serum ferritin, serum albumin) at admission, two weeks, and four weeks (at the end of induction).

Assessment of BMI

BMI is interpreted differently for children and teens even though it is calculated as weight ÷ height^2^. Because there are changes in weight and height with age, as well as their relation to body fatness, BMI levels among children and teens need to be expressed relative to other children of the same sex and age. These percentiles were calculated from the Centers for Disease Control and Prevention (CDC) growth charts, which were based on national survey data collected from 1963-65 to 1988-94 [17].

Weight was measured using a digital weighing scale, and the average of three repeated measures was recorded. Serum levels of albumin were estimated using the Bromocresol green method whereas serum folate and B12 levels were measured using electrochemiluminescence by Beckman AU 680. The results of weight, height, and BMI were compared with CDC normative data in children and adolescents. Table 1 represents the normal values for BMI and all biochemical parameters [18].

**Table 1:** Normal ranges of BMI and biochemical parameters BMI, body mass index; TIBC, total iron-binding capacity

Parameters	Normal range
BMI (percentile)	
Underweight	<5
Healthy weight	≥5 and <85
Risk for overweight	≥85 and <95
Overweight	≥95
Biochemical parameters	
Serum iron (mcg/dL)	60-180
Serum ferritin (ng/mL)	
Male	23-336
Female	11-306
TIBC (mcg/L)	155-355
Folate (ng/mL)	4.0-17.0
Vitamin B12 (pg/mL)	174-878
Vitamin D (ng/mL)	30-100
Calcium (mg/dl)	8.5-10.2
Phosphate (mg/dL)	2.5-4.5

Statistical analysis

Data was analyzed using Statistical Package for the Social Sciences (SPSS) version 21.0 (IBM Corp., Armonk, NY). Descriptive data was expressed as median with interquartile range for continuous data or as frequency (percentage) for categorical data. The chi-square test or Fisher exact test was used to compare categorical data. Continuous variables were compared using the Mann-Whitney U test for two-group comparisons and the Kruskal-Wallis test for multiple-group comparisons, both of which are non-parametric tests. P value <0.05 was considered statistically significant.

## Results

A total of 64 patients were enrolled, and the majority of them were male (67.18%). Most of the study population belonged to the age group of 16-20 years (39.06%). Patients were classified as underweight (45.31%), healthy weight (51.56%), and overweight (3.13%) based on their nutrition status according to BMI. Thirty-eight patients (59.37%) were clinically diagnosed with B-ALL. Hyperleukocytosis was present among 28% of the patients. At baseline, the low values of serum albumin, folate, vitamin B12, serum iron, TIBC, phosphorus, serum calcium, and vitamin D were reported in 20.31%, 25%, 28.13%, 6.25%, 14.06%, 4.69%, 56.25%, and 95.31% of the patients, respectively. Approximately 85% of the patients had high serum ferritin at admission, which could be falsely elevated secondary to inflammation or infection as an acute phase reactant. No significant correlation was observed between B12, calcium, ferritin, folate, iron, phosphorus, TIBC levels at admission, and nutritional status (Table 2).

**Table 2:** Demographic and baseline characteristics Data presented as n (%). BMI, body mass index; AML, acute myelogenous leukemia; APML, acute promyelocytic leukemia; B-ALL, B-cell acute lymphoblastic leukemia; B-MPAL, B-cell mixed phenotype acute leukemia; T-ALL, T-cell acute lymphoblastic leukemia; UIBC, unsaturated iron-binding capacity; TIBC, total iron-binding capacity

Patient characteristics	Number of patients (N=64)
Age group (years)	
1-5	13 (20.3)
6-10	5 (7.81)
11-15	21 (32.81)
16-20	25 (39.06)
Sex	
Male	43 (67.18)
Female	21 (32.81)
BMI	
Underweight	29 (45.31)
Healthy weight	33 (51.56)
Overweight	2 (3.13)
Clinical diagnosis	
B-ALL	38 (59.37)
AML	13 (20.31)
T-ALL	6 (9.37)
APML	5 (7.81)
B-MPAL	2 (3.12)
Hyperleukocytosis	
Yes	18 (28.12)
No	46 (71.87)
Serum albumin (g/dL)	
Normal	51 (79.68)
Low	13 (20.31)
Folate (mcg)	
Low	16 (25.00)
Normal	48 (75.00)
Vitamin B12 (mcg)	
Low	18 (28.13)
Normal	46 (71.88)
Serum iron (µmol/L)	
Low	4 (6.25)
Normal	60 (93.75)
Serum ferritin (ng/mL)	
Normal	10 (15.63)
High	54 (84.38)
TIBC (mcg/dL)	
Low	9 (14.06)
Normal	55 (85.94)
Vitamin D (ng/mL)	
Low	61 (95.31)
Normal	3 (4.69)
Serum calcium (mg/dL)	
Low	36 (56.25)
Normal	28 (43.75)
Phosphorus (mg/dL)	
Low	3 (4.69)
Normal	61 (95.31)

Additionally, 45.31% of the patients were underweight at admission, which increased to 57.81% and 60.34% after two and four weeks of induction chemotherapy, respectively. In contrast, the percentage of patients with a healthy weight at admission (51.56%) decreased to 39.06% and 36.21% after two and four weeks, respectively.

There was a trend for worsening undernutrition in both the underweight and healthy weight groups. The mean BMI at admission was 16.71 kg/m^2^, which decreased to 16.05 kg/m^2^ and 16kg/m^2^, on weeks two and four, respectively. This decrease in BMI was statistically significant (P=0.0001). A similar decreasing trend was observed in the BMI percentile (P=0.021). There was a significant decrease in serum iron levels, with a median of 165.5 ng/mL on admission, which decreased to 126 ng/mL after two weeks (P=0.001). A significant drop in TIBC values was reported, with a median of 231.5 mcg/dL on admission decreasing to 195.5 mcg/dL at two weeks (P=0.04). There was no significant correlation between folate and vitamin B12 levels at admission with nutritional status. The serum ferritin values showed a decreasing trend after admission. Also, vitamin D levels continued to be low during the four weeks of observation. No significant variation in serum calcium values was observed at two time points (Table 3).

**Table 3:** Nutritional status on admission, week two and week four Data presented as median (range); unless otherwise specified, Kruskall Wallis non-parametric tests was used to perform multiple group comparison. ^a^P-value for comparison between data at admission and at two weeks. ^b^P-value for comparison between data at admission and at four weeks. BMI, body mass index; TIBC, total iron-binding capacity; SD, standard deviation

Parameter	Normal values	On admission	2 weeks	4 weeks	P-value
BMI (kg/m^2^), mean (SD)	18.5-24.9	16.71 (3.51)	16.05 (3.65)	(n=58) 16 (3.57)	0.0001^a^
					<0.0001^b^
BMI percentile	>5 to <85	8.95 (0.500-28.750)	1.95 (0.100-32.600)	1.55 (0.100-23)	0.021^a^
					0.0002^b^
Albumin (g/dL), mean (SD)	3.4 to 5.4	3.6 (0.56)	3.48 (0.56)	(n=57) 3.71 (0.68)	0.117^a^
					0.601^b^
B12 (mcg)	<203 (174-878)	261.5 (188.5-498.5)	-	-	
Calcium (mg/dL), mean (SD)	8.5-10.2 (<8.5)	8.36 (0.67)	8.19 (0.81)	8.19 (0.81)	0.175^a^
					0.252^b^
Ferritin (ng/mL)	Males=23-336; females=11-306	1020.75 (438.660-1650)	902.5 (384-1650)	(n=57) 821 (343-1650)	0.692^a^
					0.597^b^
Folate (mcg)	4.0 - 17.0	5.78 (4.015-9.655)	-	-	
Iron (µmol/L)	11-32	165.5 (110.500-213.500)	126 (95.500-153)	126 (89-173.750)	0.001^a^
					0.009^b^
Phosphorus (mg/dL)	2.5-4.5 (<2.5)	4.41 (1.39)	-	-	
TIB C(mcg/dL)	155-355	231.5 (174-275)	195.5 (149-235)	(n=57) 212 (163.75-261.25)	0.04^a^
					0.629^b^
Vitamin D (ng/mL)	<30 (30-100)	12.2 (7.235-15.35)	10.6 (6-14.85)	(n=57) 10.9(7.925-14.475)	0.236^a^
					0.883^b^

The overall morbidity was reported in 32 patients (50%). The underweight category had significantly higher morbidity than healthy weight (65.52% vs. 39.39%; P=0.043). Fungal pneumonia was reported in 22 patients, out of which 14 patients were underweight and eight patients were from the healthy weight category. Septic shock was seen in 16 patients (underweight (nine patients) and healthy weight (seven patients)). Toxicity was observed in 29 patients (45.31%). About 15.63% of recruited patients required dose reduction/interruption of chemotherapy during induction (27.9% from underweight vs. 6.06% from healthy weight; P=0.0496). The overall mortality was 20.31% (13/64). A higher mortality was observed in the patients with undernutrition compared to patients with healthy weight (31.03% vs. 12.12%; P=0.140) (Table 4).

**Table 4:** Assessment of morbidity, toxicity, and mortality in patients with poor nutritional status Data presented as n (%); Mann-Whitney non-parametric tests were used to perform two group comparisons.

Parameter	Total (N=64)	BMI percentile		P-value
		Underweight (n=29)	Healthy weight (n=33)	
Morbidity	32 (50.00)	19 (65.52)	13 (39.39)	0.043
Fungal pneumonia	22 (34.38)	14 (48.28)	8 (24.24)	0.081
Septic shock	16 (25.00)	9 (31.03)	7 (21.21)	0.477
Toxicity	29 (45.31)	13 (44.83)	16 (48.48)	0.408
Dose reduction chemotherapy	10 (15.63)	8 (27.59)	2 (6.06)	0.0496
Mortality	13 (20.31)	9 (31.03)	4 (12.12)	0.140

**Figure 1:**
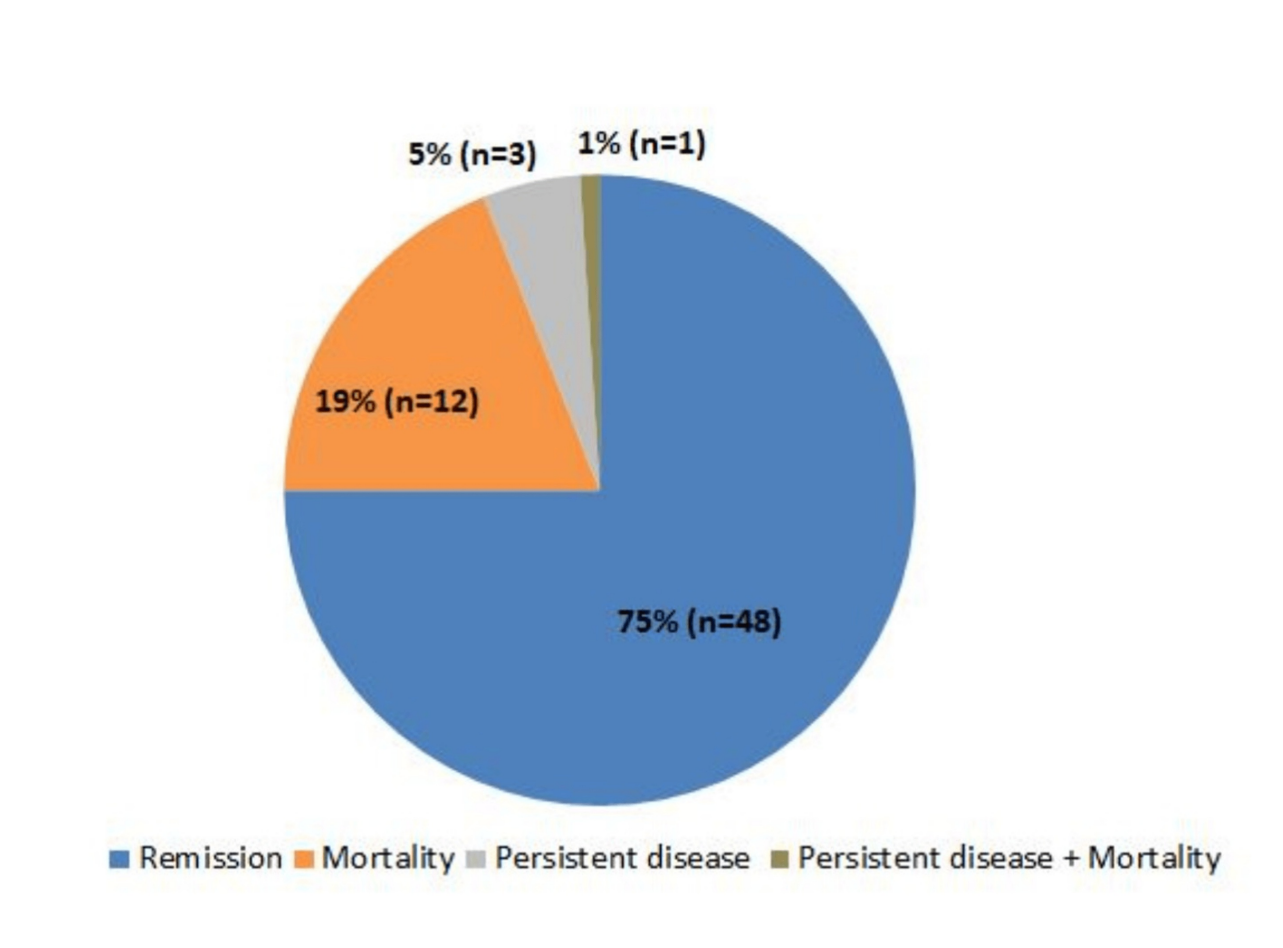
Response of patients to chemotherapy

The time between diagnosis and treatment ranged from three to 120 days, with a median of 24.5 days for patients with a poor prognosis and 20 days for others. Of all, 75% of patients achieved remission following induction chemotherapy (Figure 1).

On correlation, the serum albumin at admission and after two weeks correlated well with mortality (P<0.001) and poor prognosis (P<0.001). The median serum albumin at admission for patients with poor prognosis is 3.2 g/dL, which further declines to 2.95 g/dL at two weeks. There was no correlation between serum ferritin, serum albumin, and toxicity. Patients with high ferritin at admission had a higher chance of mortality (22.2%) when compared to those with normal ferritin (10%). There was no correlation observed between serum iron, TIBC, folate, and vitamin B12 levels at admission with toxicity or mortality.

## Discussion

For decades, the high prevalence of malnutrition among both adult and pediatric cancer patients has been appreciated and continues to be documented. While the prognostic significance of nutritional status in cancer patients remains a topic of debate, it is widely acknowledged that nutritional support is a crucial component of medical therapy [19]. The present study was designed to assess the nutritional status of children and adolescents with acute leukemia and the effect of induction chemotherapy on their nutritional status.

The study primarily included patients aged 16-20 years (39%), with a significant proportion in the 11-15 years age group (33%). The majority of participants were male (67%), and the most common diagnosis among the recruited patients was B-ALL (60%), followed by AML (20%). Similar results were reported in a study by Radhakrishnan et al. where males were predominant (65%) and ALL comprised 37% of diagnoses, followed by AML at 13% [20]. In the present study, 45% of the newly diagnosed acute leukemia patients were underweight based on their BMI for age, a notably higher percentage than the 15% observed in developed countries [21]. This data is consistent with earlier findings from India by Shah et al., where underweight cases accounted for 35% [22]. Brinksma et al. found that in high-income countries, individuals with leukemia had a low prevalence of malnutrition, approximately 5-10% experienced malnutrition at diagnosis, dropping to 0-5% during treatment [23]. The mean BMI percentile at admission was 20.06 kg/m^2^, which decreased to 17.6 kg/m^2^ and 15.22 kg/m^2^ at two and four weeks, respectively. As in the case of BMI, the BMI percentile also showed a similar decreasing trend. This was statistically significant (P=0.021). A similar trend was observed in the study by Gustaitė et al. [24], where high white blood cell counts at the initial presentation of acute leukemia were associated with a higher early mortality rate, often due to leukostasis. A study conducted by Oliviera et al. [25] on hyperleukocytosis demonstrated a significantly shorter overall survival (P<0.0001) and a greater incidence of early deaths (P=0.0008). An Indian study by Seth et al. also demonstrated similar findings [26]. In the current study, 28% of patients had hyperleukocytosis upon admission. While patients with hyperleukocytosis had a higher mortality rate (27.7%) compared to those without hyperleukocytosis (17.39%), this difference was not statistically significant (P=0.353). Results from a study by Kittivisuit et al. reported similar findings to the present study [27].

Chemotherapy and recurrent episodes of sepsis may deplete the body of the essential macro- and micronutrients needed for appropriate growth and metabolism [28]. Serum albumin is a commonly used nutritional index in clinical practice. However, its extended half-life and susceptibility to changes due to stress and illness make it a non-specific measure of nutritional status. Additionally, pro-inflammatory mechanisms, often linked to tumors, can impact liver function and, consequently, albumin synthesis. In the present study, 20% of patients had low baseline albumin levels (<3.5 mg/dL). While there was a trend of decreasing albumin levels from admission to two weeks, this change was not statistically significant (P=0.117). However, lower albumin levels at admission and at two weeks were associated with a poorer prognosis (mortality + persistent disease), with significant differences observed (P=0.001). There was no correlation found between serum albumin and drug-related toxicity. In a study by Tandon et al. [12], deficiencies in vitamin B12 and folate were significantly linked to toxic deaths during induction chemotherapy. In this study, around 25% of patients had low folate and B12 levels at admission. However, no correlation between these deficiencies and drug-related toxicity or mortality was observed in the present study population. Previous studies suggested that elevated serum ferritin levels in AML patients are the result of dysregulations in the inflammatory response, chemotherapy, and disease burden, which is released by leukemic cells. Hence, serum ferritin may act as an inflammatory marker, which may affect disease progression [29]. A study by Yokus et al. [30] assessed iron overload in patients with acute leukemia and reported elevated serum ferritin levels from baseline to the first month in both AML and ALL patients. However, in the present study, the average serum ferritin level was 1020.75 ng/mL, which declined to 902.5 ng/mL at two weeks and 821 ng/mL at four weeks, with no statistically significant difference observed.

In the current study, febrile neutropenia was observed in 44 out of 64 patients (68%) during induction chemotherapy. In a previous study by Prasoon Sebastian et al. [31], which included 44 patients with acute leukemia treated with intensive chemotherapy, febrile neutropenia developed in all patients undergoing AML induction therapy and in 21.4% of those receiving induction therapy for ALL. Morbidity, which included fungal pneumonia, necrotizing enterocolitis, and sepsis/septic shock, was observed in 50% of the patients (n=32). The underweight category had a significantly higher morbidity rate of 65.52% compared to 39.39% in those with a healthy weight, as supported by statistical significance (P=0.043). Fungal pneumonia affected 34.38% of the recruited patients. Among underweight patients, 48.28% had fungal pneumonia, which is quite high (compared to fungal rates in developed countries). Septic shock occurred in 25% of the patients. In the underweight group, 31.03% of patients experienced septic shock. In a study conducted by Rimjhim Sonowal et al., which included 101 pediatric patients with ALL undergoing induction chemotherapy, the findings showed that out of all included patients, 52.5% of patients had acute undernutrition at diagnosis. Among ALL patients with acute undernutrition, the incidence of severe infection was found to be 10.8% higher than that in ALL patients with normal nutrition, but this difference was statistically insignificant [32]. In a study by Kumar et al., the observed difference in infection rates between well-nourished and malnourished children during the induction phase of treatment shows a trend toward significance (p=0.06) [33].

In the present study, a significant difference was observed in the interruption or dose reduction of chemotherapy between underweight and healthy-weight patients. Specifically, 27.9% of underweight patients experienced such interruptions or reductions, compared to only 6.06% of those with a healthy weight. Similar findings were reported by Roy et al., where a significant proportion of patients (61.63%) were unable to complete chemotherapy within the specified 145 days, and 76.67% of these patients belonged to the severe malnutrition group (P<0.002). Also, a declining trend was observed in achieving remission after induction chemotherapy with increasing levels of undernutrition [34]. In this study, 75% of patients achieved remission following induction chemotherapy, while the remaining 25% did not (comprising mortality and persistent disease). Notably, patients with undernutrition had a higher mortality rate of 31%, compared to 12% in those with a healthy weight. However, this difference was not statistically significant. A study by Flegal et al. and Linga et al. have reported findings similar to the present study [13,35]. Generally, commercially available oral nutritional supplements are recommended for children to manage malnutrition. Additionally, infusions of supplemental immunoglobulin and prophylactic antibiotics are advised for undernourished children with AML undergoing chemotherapy [36]. However, further research is needed to evaluate the long-term outcomes of nutritional supplementation in patients with acute leukemia.

Limitations

The study's findings may not apply to the broader population as it focused on economically disadvantaged patients in a government center's general wards. Patient heterogeneity made it challenging to analyze all cancer-related factors. Nutritional status was assessed during induction chemotherapy, but post-discharge evaluations were missing, and long-term follow-up could offer more insights. The study did not explore the long-term effects of nutritional status on outcomes, relapse, or chronic complications.

## Conclusions

In the present study, undernourished patients demonstrate a significant inclination toward elevated morbidity. Moreover, it is noteworthy that there was a rise in mortality rates among these patients in comparison to others, although this disparity did not attain statistical significance (P=0.140). It is crucial to highlight the impact of low albumin levels, as they have been associated with increased mortality in similar patient populations. Furthermore, underweight patients experienced more frequent dose interruptions or reductions in chemotherapy. Therefore, a thorough assessment of nutritional status upon admission for acute leukemia patients is imperative. Future research should focus on exploring the potential advantages of supplemental nutrition and elucidating the interplay between chemotherapeutic agents and nutritional status in larger prospective trials.
